# Enterotoxigenic *Escherichia coli* Multilocus Sequence Types in Guatemala and Mexico

**DOI:** 10.3201/eid1601.090979

**Published:** 2010-01

**Authors:** Matilda Nicklasson, John Klena, Claudia Rodas, August Louis Bourgeois, Olga Torres, Ann-Mari Svennerholm, Åsa Sjöling

**Affiliations:** University of Gothenburg, Gothenburg, Sweden (M. Nicklasson, C. Rodas, A.-M. Svennerholm, Å. Sjöling); United States Naval Medical Research Unit 3, Cairo, Egypt (J. Klena); Johns Hopkins Bloomberg School of Public Health, Baltimore, Maryland, USA (A.L. Bourgeois); Instituto de Nutrición de Centro América y Panamá, Guatemala City, Guatemala (O. Torres)

**Keywords:** MLST, CS6, Escherichia coli, heat-stable toxin, sequence type, bacteria, Guatemala, Mexico, dispatch

## Abstract

The genetic backgrounds of 24 enterotoxigenic *Escherichia coli* (ETEC) strains from Mexico and Guatemala expressing heat-stable toxin (ST) and coli surface antigen 6 (CS6) were analyzed. US travelers to these countries and resident children in Guatemala were infected by ETEC strains of sequence type 398, expressing STp and carrying genetically identical CS6 sequences.

Enterotoxigenic *Escherichia coli* (ETEC) is one of the most common causes of acute watery diarrhea among children and adults in the developing world, causing ≈400 million diarrheal episodes and 380,000 deaths in children <5 years of age every year (*1*). The diarrhea is mediated by 1 or 2 plasmid-encoded enterotoxins; the heat-stable toxin (ST) and/or the heat-labile toxin (LT) (*1*). Approximately one third of all ETEC strains isolated globally reportedly produce ST only, one third produce LT and ST, and one third produce LT only (*2*). Two genotypes of ST exist in ETEC strains infecting humans: STp and STh (*3*). Colonization of the small intestine is mediated by adhesion to the epithelial cells by colonization factors (CFs), and one of the most common CFs detected on clinical ETEC isolates from different parts of the world is the plasmid-encoded coli surface antigen 6 (CS6) (*4*).

In a recent vaccine trial conducted in Mexico and Guatemala involving adult US travelers, ST-only strains expressing only CS6 and no other CF predominated among those infected with CF-positive ETEC; this toxin-CF profile (ST/CS6) was present in 35% of diarrheal cases (*5*). In other geographic regions, e.g., Egypt and Bangladesh, studies on childhood diarrhea have reported ST/CS6 frequencies of 6.6% and 19%, respectively (*6*,*7*). We conducted this study to investigate whether adult travelers in Mexico and Guatemala are infected with certain circulating ST/CS6 ETEC strains and to determine whether ETEC strains with the same *E. coli* clonal background may be infecting resident children in the same areas.

## The Study

Seventeen clinical ST/CS6 isolates collected from adult US travelers, who were either visiting various locations in Guatemala or remaining at least 14 days in Antigua in Guatemala, or in Cuernavaca, Mexico, were included in the study. Isolates were collected from 1998 to 2001 during ETEC vaccine trials (*5*,*8*) or in 2002 and 2003 during a study of antimicrobial drug treatment (*9*). During June 2001–October 2003, clinical stool specimens were collected from children living in Santa María de Jesús in Guatemala as part of an ongoing childhood ETEC study (O. Torres, unpub. data). Seven CS6 isolates obtained from that study, collected in the summer of 2002, were included in the present study. Clinical isolates from both children and adults were shipped to Sweden, where toxin and CF profiles were confirmed as described (*10*). All strains in the study expressed STp, except for strain E874, which expressed STh.

Isolates were analyzed by multilocus sequence typing (MLST) by using the *E. coli* MLST scheme (http://mlst.ucc.ie/mlst/dbs/Ecoli), which is based on sequencing of internal regions of the 7 housekeeping genes *adk, fumC, gyrB, icd, mdh, purA,* and *recA* (*11*). PCR was performed as described but by using the same annealing temperature (54°C) for all genes.

Seven MLST sequence types were observed among the 24 ST/CS6 ETEC isolates (Table). The most common were MLST sequence type 398 (ST-398) (n = 10 [all from Guatemala]), sequence type 182 (n = 6 [4 from Guatemala, 2 from Mexico]), and sequence type 278 (n = 4 [2 each from Guatemala and Mexico]). Three novel MLST sequence types (all from Guatemala) were identified and, upon submission to the *E. coli* MLST database, were designated as MLST sequence types 712, 726, and 727. Sequence type 726 clustered closely with the sequence type 182 isolates (Figure); these 2 sequence types are single locus variants, differing only in *mdh* (mdh-6 v mdh-1). Sequence type 727 is a single locus variant of sequence type 278, differing only in *gyrB* (gyrB-1 v gyrB-33). Sequence type 712 is a single locus variant of sequence type 398, differing only in *fumC* (fumC-23 v fumC-7).

**Table:** Comparison of ETEC isolates and clinical and demographic information collected during study of US travelers to Guatemala and Mexico and resident children from Guatemala*

Isolate†	Toxin/CF profile	Geographic origin	Date of collection	Severity of diarrhea‡	Age/sex	MLST sequence type
Travelers						
E617	STp/CS6	Antigua, Guatemala	2000 Jun 19	Mild	24 y/M	182
E830	STp/CS6	Traveling in Guatemala	2002 Sep 23	Moderate–severe	30 y/M	182
E539	STp/CS6	Cuernavaca, Mexico	2000 Jul 13	Moderate–severe	36 y/F	182
E576	STp/CS6	Cuernavaca, Mexico	2000 Jul 15	Moderate–severe	50 y/M	182
E494	STp/CS6	Antigua, Guatemala	1999 Jul 3	Moderate–severe	37 y/F	182
E695	STp/CS6	Antigua, Guatemala	2001 Jan 15	Moderate–severe	44 y/M	182
E396	STp/CS6	Antigua, Guatemala	1998 Jul 30	Moderate–severe	49 y/M	726
E848	STp/CS6	Traveling in Guatemala	2003 Apr 28	Moderate–severe	27 y/M	727
E368	STp/CS6	Cuernavaca, Mexico	1999 Aug 11	Asymptomatic§	30 y/F	278
E416	STp/CS6	Antigua, Guatemala	1999 May 30	Moderate–severe	25 y/M	278
E521	STp/CS6	Cuernavaca, Mexico	2001 Apr 8	Moderate–severe	51 y/F	278
E837	STp/CS6	Traveling in Guatemala	2002 Sep 23	Moderate-severe	30 y/M	278
E844¶	STp/CS6	Traveling in Guatemala	2003 Apr 28	Moderate–severe	29 y/F	712
E382	STp/CS6	Antigua, Guatemala	1998 Jul 1	Moderate–severe	21 y/F	398
E447	STp/CS6	Antigua, Guatemala	1999 Jun 21	Moderate–severe	46 y/F	398
E670	STp/CS6	Antigua, Guatemala	2000 Jul 17	Moderate–severe	27 y/F	398
E850¶	STp/CS6	Traveling in Guatemala	2003 May 8	Asymptomatic	29 y/F	398
Children						
E856	STp/CS6	SMJ, Guatemala	2002 Jun 21	Persistent moderate–severe	10 mo/M	398
E861	STp/CS6	SMJ, Guatemala	2002 Jul 4	Persistent moderate–severe	34 mo/M	398
E870	STp/CS6	SMJ, Guatemala	2002 Jul 26	Moderate	11 mo/M	398
E871	STp/CS6	SMJ, Guatemala	2002 Aug 1	Moderate	22 mo/F	398
E872	STp/CS6	SMJ, Guatemala	2002 Aug 7	Moderate	12 mo/F	398
E879	STp/CS6	SMJ, Guatemala	2002 Aug 23	Moderate	26 mo/F	398
E874	STh/CS6	SMJ, Guatemala	2002 Aug 19	Moderate	18 mo/F	443

*ETEC, enterotoxigenic *Escherichia coli*; CF, colonization factor; MLST, multilocus sequence typing; SMJ, Santa María de Jesús. †World Health Organization Collaborating Centre for Research on Enterotoxigenic *Escherichia coli* strain collection number. ‡Classification of diarrheal disease severity in travelers was based on the study case definition of travelers’ diarrhea with an associated gastrointestinal symptom rated as mild, moderate, or severe (*5*). Moderate symptoms interfered with daily activity, and severe symptoms prevented normal daily activity. Disease classification in children was based on the number of diarrheal stools in a 24-hour period (3/24 h, mild; 4–5/24 h, moderate; >6/24 h, severe). Diarrhea accompanied by vomiting was classified as severe; diarrhea lasting >14 days was classified as persistent. §Not travelers’ diarrhea. ¶Strains E844 and E850 were isolated from the same person participating in the antimicrobial drug treatment study. Strain E850 was isolated after completion of a course of antimicrobial drugs.

**Figure:**
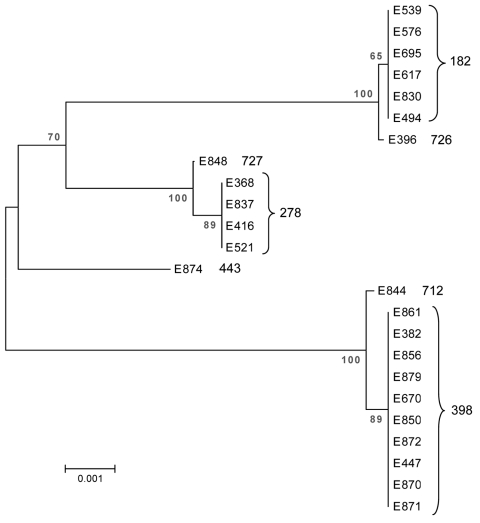
Dendrogram of the 24 enterotoxigenic *Escherichia coli* strains from Guatemala and Mexico included in the study, showing multilocus sequence type. Sequences were assembled with BioEdit and aligned using ClustalX within BioEdit (*12*). The dendogram represents the relationship of a concatenation of the sequences from each strain and was constructed by using MEGA 3.1 (*13*). Phylogenetic reconstructions were created by using the neighbor-joining method with the Kimura 2-parameter substitution model, using 1,000 bootstrap replicates. A similar arrangement of the strains was indicated by eBURST version 2 analysis (http://eburst.mlst.net). Scale bar indicates dissimilarity, where 0 is completely identical and 1 is completely dissimilar.

The single STh/CS6 strain in the study, E874 from a child, was the only representative for MLST sequence type 443. This strain did not cluster closely with any of the other isolates by MLST (Figure). The 6 remaining isolates from the childhood study (all STp/CS6) clustered into sequence type 398. Three isolates from adult travelers collected in Antigua in Guatemala during 1998, 1999, and 2000 (E382, E447, and E670) and 1 collected from a traveler in Guatemala in 2003 (E850) also clustered into sequence type 398. The remaining adult travelers were infected by ETEC strains that clustered mainly into sequence types 182 (n = 6) and 278 (n = 4) (Table; Figure); these sequence types were found in Mexico and Guatemala during 1999–2002. Two of the strains, E830 (sequence type 182) and E837 (sequence type 278), were isolated from the same person on the same day, highlighting the possibility for several pathogens to simultaneously cause diarrhea.

A 540-bp internal region of the CS6 operon (*cssABCD*) encompassing the distal part of *cssB*, an intergenic untranslated region, and the proximal part of *cssC*, contains sequence differences in LT-only strains (*14*). Sequencing of the same region (performed as described [*14*]) in the 24 ST-only isolates in this study showed that all STp/CS6 isolates had identical CS6 sequences to the STp/CS6 ETEC strain GB124 (GenBank accession no. DQ538390, nt 815–1354) (*14*), isolated in Guinea Bissau. On the other hand, the only STh/CS6 isolate in the study, E874, had a different CS6 sequence with 7 nonsynonymous and 4 synonymous nucleotide substitutions compared with the other strains. The CS6 sequence of E874 was most similar to the CS6 sequence of the STh+LT–positive strain E10703 (GenBank accession no. U4844) (*15*), with only 2 adjacent nucleotide differences, resulting in a Q to R substitution at nt 139 of *cssC* in strain E874.

## Conclusions

US travelers to Mexico and Guatemala were infected by strains of 6 different MLST sequence types, showing that the high prevalence of ST/CS6 among travelers in this region is not due to infection by a single circulating ST/CS6 ETEC strain. Results also showed that adult travelers and resident children may be infected by ETEC strains with the same *E. coli* genetic background because ST/CS6 strains of MLST sequence type ST-398 infected both adult travelers and resident children in Guatemala. This MLST sequence type circulated in Guatemala for at least 6 years, indicating the presence of persistent MLST sequence types.

All STp/CS6 strains in the study had identical sequences in a region of the CS6 operon previously found to vary greatly in LT-only strains (*14*), even though the STp/CS6 strains represented 6 different MLST sequence types and were collected over 6 years and at different sampling sites in Guatemala and Mexico. This sequence was identical to the corresponding CS6 sequence in an STp/CS6 strain from Guinea-Bissau (*14*) but differed from the available sequences of ST/LT and LT-only CS6 strains (GenBank accession nos. UO4844, UO4846). This finding may indicate that plasmids carrying the genes for STp and CS6 are genetically conserved and have spread across the world because identical sequences were found in northern Latin America and in West Africa. Further studies have therefore been initiated to explore the genetics and epidemiology of ETEC strains expressing CS6 and STp in a global perspective.
